# Long-Term Performance Evaluation of an FRP Composite Road Bridge Using DFOS Monitoring System

**DOI:** 10.3390/s25237131

**Published:** 2025-11-21

**Authors:** Maciej Kulpa, Tomasz Siwowski, Mateusz Rajchel, Ewa Błazik-Borowa, Michał Jukowski

**Affiliations:** 1 Department of Road and Bridges, Rzeszow University of Technology, 35-084 Rzeszów, Poland; siwowski@prz.edu.pl (T.S.); mrajchel@prz.edu.pl (M.R.); 2 Department of Structural Mechanics, Lublin University of Technology, 20-618 Lublin, Poland; e.blazik@pollub.pl; 3 Department of Road and Bridges, Lublin University of Technology, 20-618 Lublin, Poland; m.jukowski@pollub.pl

**Keywords:** FRP bridge, visual inspection, structural health monitoring, load test, distributed fibre optic sensing, safety, stiffens, dynamic characteristics, repair

## Abstract

FRP composite bridges have been in operation since the mid-1990s, allowing for the evaluation of their long-term behaviour. Many of the early FRP bridges in the USA and Western Europe were equipped with monitoring systems to assess their structural integrity after years of use. In Poland, the first all-FRP composite bridge was also equipped with a modern structural health monitoring (SHM) system based on distributed fibre optic sensing (DFOS) to enable long-term performance monitoring. Over nearly a decade of use, the bridge’s strain, stiffness, and dynamic properties have been evaluated three times through static and dynamic load tests. Research findings indicate that the bridge has maintained satisfactory structural integrity and durability over an eight-year operational period. However, the quality of the adhesive joints between the girders and the deck panels was found to be inadequate, resulting in a slight decrease in the bridge’s performance, specifically in stiffness and dynamic characteristics. Fortunately, these negative changes did not compromise the bridge’s safety or serviceability, as stipulated by the design requirements. An effective repair was completed, restoring the bridge to its full operational efficiency.

## 1. Introduction

Since the early 1980s, fibre-reinforced polymer (FRP) composites have been utilized in various bridge applications due to their exceptional strength, lightweight nature, and durability. These materials can also be tailored to meet the specific requirements of complex designs [1,2,3,4,5,6,7]. Today, FRP composite technology has advanced from laboratory prototypes to actual demonstration projects in the field. FRP composites are primarily employed in the construction of large-scale structures, such as deck systems, footbridges, and vehicle bridges [8,9,10,11]. The numerous advantages of FRPs encourage bridge owners, designers, and contractors to incorporate this material into different structural forms. The benefits can be summarized as follows: weight savings (due to their high strength-to-weight ratio), low maintenance requirements, resistance to environmental impacts (corrosion-free), the capability to be shaped into complex forms, and the ease of installation for off-site engineered and fabricated elements. Despite the fact that composite structures have been built for decades, their durability and potential degradation over time are still not fully understood. Observations conducted on actual structures and conclusions drawn from maintenance work are the best source of knowledge about this type of construction.

The existing all-FRP composite bridges have been in operation as full-load-bearing superstructures since the mid-1990s. In some instances, these bridges have been in service for over 30 years, allowing for an assessment of their long-term behaviour. Many of the early bridges were equipped with monitoring systems to evaluate their structural integrity after years of use. Throughout their service life, static and dynamic field load tests have been conducted on all-FRP composite bridges multiple times. By comparing the strains, displacements, and dynamic characteristics measured over time, conclusions can be drawn regarding the long-term performance of the bridges during specific periods. The results of these field tests are now becoming available, providing valuable insights into the durability and performance of all-FRP composite bridge structures [12,13,14,15,16,17,18,19,20,21]. An overview of several published results from these studies enables the evaluation of various all-FRP composite bridges after different periods of operation.

Based on the review of studies [12,13,14,15,16,17,18,19,20,21], it can be concluded that all-FRP composite bridges have shown impressive durability, structural integrity, and environmental stability over a 30-year service period. No severe damage or structural degradation has been observed in the FRP structures. The data indicates that there have been insignificant changes over the long term in terms of strength, stiffness, dynamic performance, and creep effects. In some cases, there has also been minimal variation in the composite action between the main beams and the deck. Field load testing has revealed that these all-FRP composite bridges have not encountered any structural issues and have been performing well in service. Overall, the all-FRP composite bridges have demonstrated satisfactory performance reliability during the years monitored and can be considered reliable.

The maximum recorded strains in the follow-up tests were slightly higher than those observed during the initial proof load tests. However, they were significantly lower than the predicted values and considerably less than the approximate failure strains of FRP composites. This indicates a much larger load capacity and results in a very high safety factor regarding strength. The measured deflections showed no significant difference in stiffness and were well below the design deflection limit of L/800. It is important to note that the stiffness of all-FRP composite bridges may be influenced by temperature. Nonetheless, the overall difference in maximum temperature response was less than the impact of a single truckload on the bridge. Additionally, the impact factors, natural frequencies, and damping ratios indicated that all-FRP composite bridges were performing well in service without any structural issues. Finally, the measured creep caused by dead load corresponded well with predictions from published creep data, making it easily predictable.

In Poland, the construction of bridge structures using load-bearing elements such as girders and decks made from FRP composites began in 2015. This initiative followed two research and development projects led by Rzeszów University of Technology and Gdańsk University of Technology, which involved manufacturers and contractors specializing in composite structures [22,23,24]. One of the earliest projects from this initiative was an all-FRP composite road bridge built in Rzeszów in 2016 [25,26]. Similarly to early bridges in the USA and Western Europe, this Polish all-FRP composite bridge was equipped with a modern structural health monitoring (SHM) system to enable long-term monitoring of its health and performance during service. Of the many systems currently being developed that allow for the observation and monitoring of structures [27,28], in this case it was decided to use system based on distributed fibre optic system. Over nearly a decade of use, the bridge’s strain, stiffness, and dynamic properties have been evaluated three times through static and dynamic load tests. This article compares these assessments and provides insights into the long-term performance of Poland’s first all-FRP composite bridge.

## 2. Description of the First Polish All-FRP Composite Road Bridge

**Figure 1:**
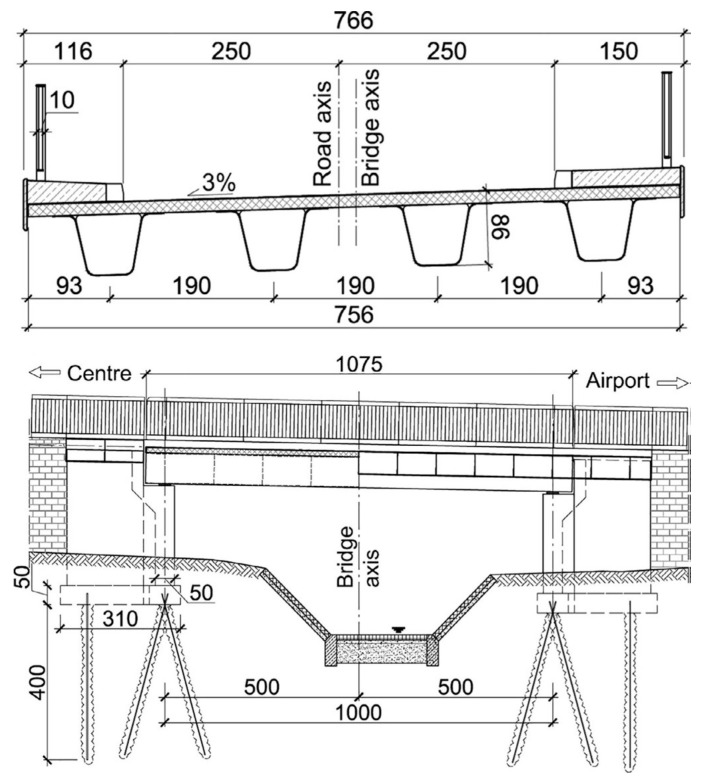
Cross-section of the all-FRP composite superstructure (**top**) and side view/longitudinal section (**bottom**).

The first all-FRP (fibre-reinforced polymer) composite road bridge in Poland is located in Rzeszów. It spans an urban road over a small local stream and has a total length of 10.7 m. This single-span bridge features a deck that is 7.7 m wide, accommodating a roadway that is 2 m wide on each side, along with two sidewalks measuring 0.75 m and 1.1 m in width, respectively (Figure 1). According to Polish bridge standards, its nominal carrying capacity is 300 kN. The superstructure of this all-composite bridge consists of four FRP composite girders, which support a 0.13-m-thick sandwich deck slab made of FRP. The deck slab is securely bonded to the top flanges of the girders using epoxy adhesive. Additionally, the deck includes two lightweight concrete sidewalk slabs reinforced with GFRP (glass fibre-reinforced polymer) bars, surrounded by stone curbs and polymer cornice plates. It also features a thin insulation layer, a pavement layer, two expansion joints, and steel balustrades. Eight elastomer bearings support the FRP composite superstructure on the bridge’s abutments. The solid reinforced concrete (RC) abutments sit on ten micropiles, each with a diameter of 110 mm and a length of 4.0 m [25].

The FRP girders feature a U-shaped cross-section with slightly inclined webs. They have two top flanges, each measuring 220 mm in width and 15 mm in thickness, as well as a bottom flange that measures 340 mm in width and 15 mm in thickness. The girder has a maximum width of 1380 mm and a depth of 715 mm. It features top and bottom flanges made of solid GFRP composites, while the webs are constructed as sandwich panels with a PVC foam core between two layers of GFRP face sheet laminates. To improve the torsional stiffness of the FRP girder and to prevent web buckling, nine internal diaphragms are spaced along its length. These diaphragms consist of 46 mm-thick sandwich, similarly to the girder’s webs. The sandwich deck panels have two 12 mm-thick GFRP laminate face sheets and a 105 mm-thick PUR foam core, which is supported by internal vertical GFRP ribs. For reinforcing the FRP composite superstructure, unidirectional and biaxial stitched glass fabrics were used, with a unit weight that varies from 800 to 1200 g/m^2^. The core material for the sandwich components are PVC foams with a density of 80 kg/m^3^ for girders and PUR foam with a density of 105 kg/m^3^ for deck slab. The composite superstructure was manufactured using VARTM infusion technology, utilizing epoxy resin as the matrix for all composite elements [25].

**Figure 2:**
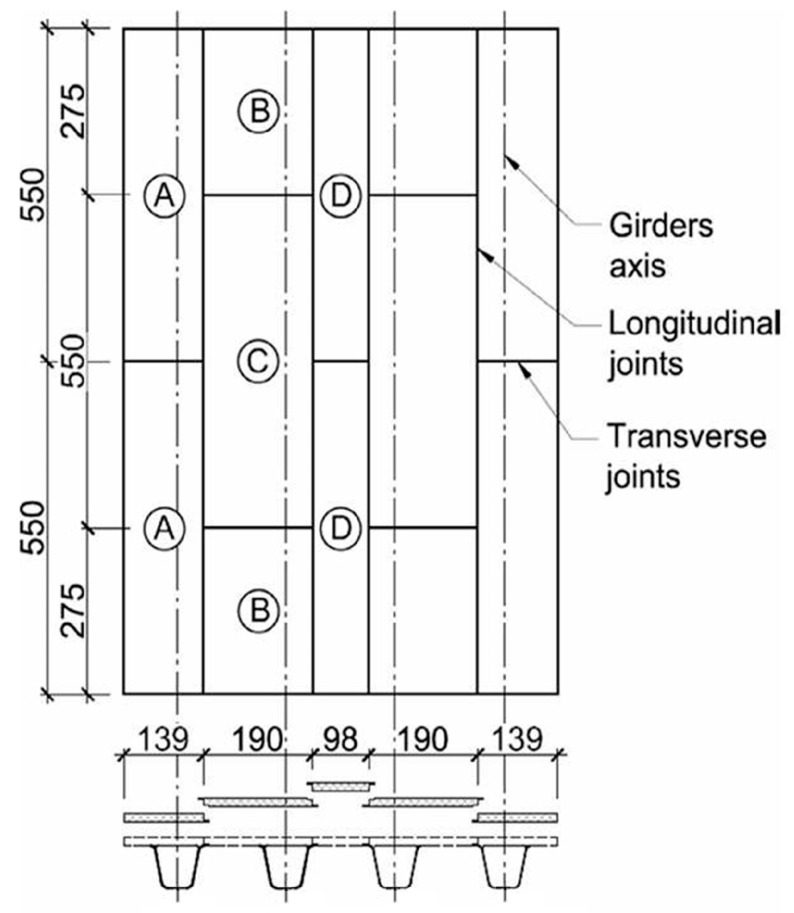
FRP superstructure assembly parts and locations of their bonded joints.

To improve the efficiency of manufacturing, transporting, and assembling the entirely composite superstructure, it was divided into several parts for fabrication and assembly. Each girder was produced through a single infusion process. Due to manufacturer constraints, the sandwich deck slab was fabricated in a total of 12 panels, consisting of four different types (A to D) and dimensions (Figure 2). Individual transverse and longitudinal adhesive joints were developed and tested to facilitate the assembly of all panels into a single deck slab [29]. Some of these joints were completed in the workshop, while others were completed on site. Two sections of the bridge superstructure were assembled in the workshop. The A, B, and C panels from one side of the deck, along with two corresponding girders, were assembled together by connecting all components with epoxy adhesive. Once the adhesive joints cured, the two superstructure tandems were transported to the site and placed on bearings that had been previously installed on the reinforced concrete abutments. Finally, the D closing panels were affixed to the deck using adhesive, and the bridge equipment was installed on top of the completed superstructure.

## 3. Visual Inspection and Repair of Damages

Damages in FRP bridges are inevitable due to the long-term effects of both static and dynamic loads. Therefore, structural health monitoring of these bridges is crucial. This monitoring should involve two sequential categories of methods. First, simple techniques such as visual inspection are employed to identify potential damage. Following this initial assessment, more advanced techniques like load tests are conducted to ascertain the type and extent of the damage. Visual inspection is the simplest and most commonly used method for bridge assessment. It can quickly reveal damage to the bridge structures when combined with basic tools such as flashlights, rulers, and mirrors. However, the limitation of visual inspection is that it only detects surface damage; it cannot quantify the severity of the damage or identify internal defects within the structures. Therefore, when visual inspections reveal signs of damage—such as cracks, delamination, discoloration, holes, and deformation—it is essential to conduct further testing using more precise methods [4,30].

**Figure 3:**
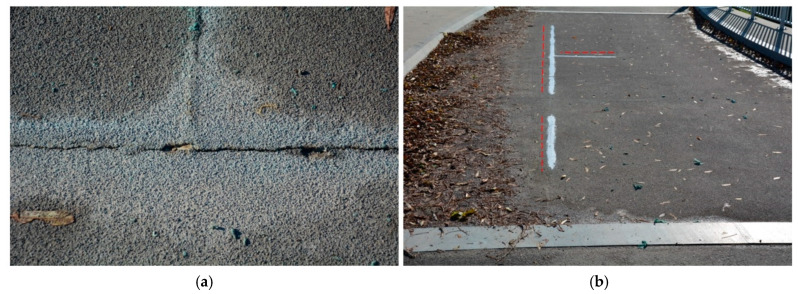
Damages observed in 2017 (cracks: a total length of approx. 8 m). (**a**) Close-up of the cracks. (**b**) Sealing of cracks.

Annual visual inspections and bridge load tests, scheduled every five years, have been established as a method for monitoring the bridge’s condition. To facilitate the recording of load test results, a specialized measuring system was installed on the all-FRP composite structure. However, during the first visual inspection conducted just a few months after the bridge’s opening (2017), damage was observed: cracks were found in the pavement along the adhesive assembly joints of the deck panels (Figure 3a). These cracks measured a maximum opening of 2 mm and had a total length of approximately 8 m. To prevent further damage during the upcoming winter and to mitigate the risk of water infiltrating the FRP structure during freeze–thaw cycles, an emergency repair was undertaken, which included sealing the surface cracks with a durable plastic material (Figure 3b).

**Figure 4:**
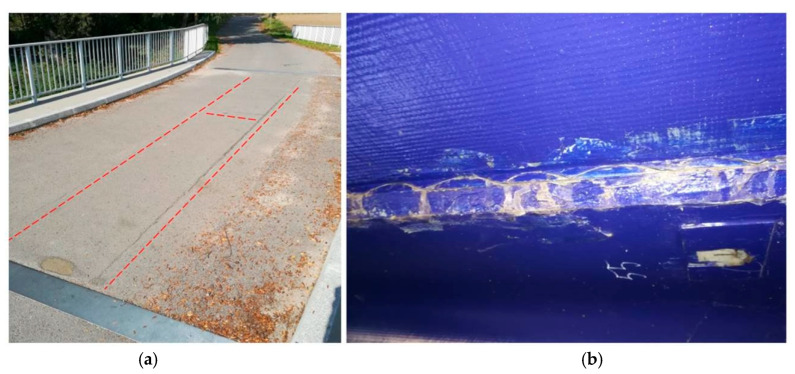
Damages observed in 2018 (cracks: a total length of approx. 22 m). (**a**) Location of surface cracks. (**b**) Cracks on the adhesive surface.

**Figure 5:**
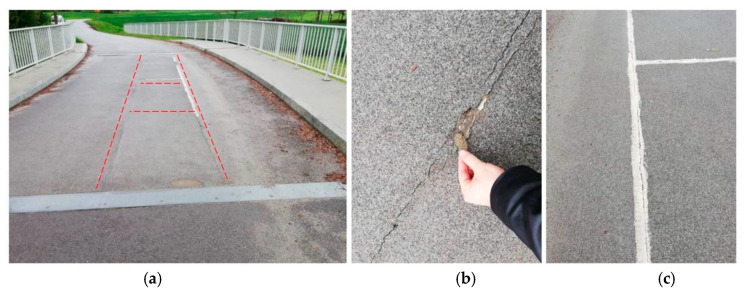
Damages observed in 2019 (cracks: a total length of approx. 24 m). (**a**) Location of surface cracks. (**b**) Close-up of the cracks of surface. (**c**) Sealing of cracks.

The surface sealing was ineffective in preventing the propagation of existing cracks. By 2018, the total length of cracks in the deck slab had reached approximately 22 m, extending nearly the entire length of the joints that were made during on-site assembly (see Figure 4a). The seal applied in 2017 also lost its effectiveness, as new cracks began to appear at the edges of the sealing material. Additionally, cracks were noted on the surface of the adhesive assembly joint between the girder and the deck slab. Due to the nature of this adhesive connection, which is concealed beneath the deck, it was not possible to determine whether there was any local separation between the deck slab and the main girder (see Figure 4b). No repairs were conducted for the following few months. The condition of the bridge stayed largely the same until 2019 (Figure 5a). By that time, cracks had developed along the entire 24-m length of the joints made during construction, and both the seals beneath the surface and the temporary seals installed in 2017 were no longer effective (Figure 5b,c).

Due to the poor condition of the surface and the risk of further water penetration into the structure, it was decided to repair the superstructure. The joints were cleaned, and loose material was removed from above. Separation between the bridge deck and the girder was observed near the supports, where the largest crack opening in the road surface was also found. In this area, the joints were repaired by injecting adhesive under pressure. In locations where the adhesive was reapplied, mechanical connectors were added between the deck slab and the upper flange of the girder to ensure proper connection while the adhesive cured. In the damaged area, a thin layer of surface material was reapplied, and the thin-layer surface on the roadway was also renewed.

**Figure 6:**
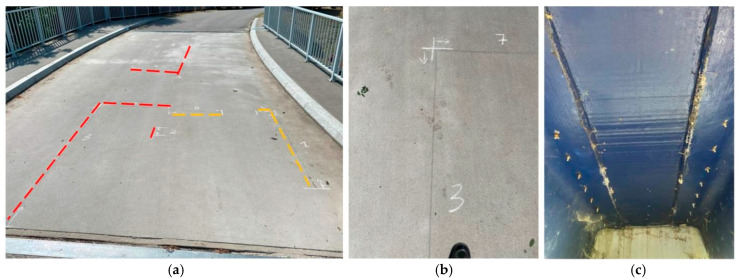
Damages observed in 2024. (**a**) Cracks: a total length of approx. 11 m; in red—cracks partially reopened after repairing; in orange—cracks showed after repairing in 2019). (**b**) Sharp and clear ending of the cracks on the corner of the deck panel. (**c**) View of the completed reinforcement of the panel-to-girder connection.

In 2024, a decision was made to conduct a subsequent load test to assess the current condition of the structure and its response to the proof load. This test aimed to verify the condition of the superstructure after repairs had been completed. Prior to loading the structure, the bridge underwent an inspection, which revealed that cracks had reappeared on the deck slab. Most of these cracks followed the original patterns, but new cracks also emerged in the joints that were fabricated in the workshop. Meanwhile, the joints constructed on-site, which had previously exhibited cracks along their entire length, are now shorter due to the reapplication of glue in 2019. Currently, the road surface is in significantly better condition than it was prior to the repairs. It was noted that the cracks terminate sharply, without extending into the adjacent deck panels (see Figure 6b). In the areas where glue was reapplied and mechanical fasteners were added, no crack propagation was observed (see Figure 6c).

## 4. Bridge Load Tests

### 4.1. Test Objectives and Schemes of Test Loading

To verify the design assumptions and identify the basic dynamic characteristics of the bridge, as well as to monitor its behaviour during operation, two proof load tests of the all-FRP composite superstructure were conducted under controlled load conditions. The first test took place immediately after the bridge’s completion and before it was opened to traffic. The second test was performed under the same loading conditions after eight months of service, during which traffic was halted for a few hours to facilitate the testing process. This testing schedule was expanded in 2024, with additional follow-up tests conducted to assess the effectiveness of repairs made to the FRP superstructure. In total, three load tests of the bridge were carried out between 2016 and 2025, according to the following schedule:

- 2016: proof load test before the bridge opening to check design assumptions and verify dynamic characteristics;
- 2017: proof load test before the end of the warranty period to evaluate the bridge’s behaviour after one year of operation;
- 2024: follow-up load test to assess the effectiveness of the bridge repair and to check the current state-of-repair of the bridge.

**Figure 7:**
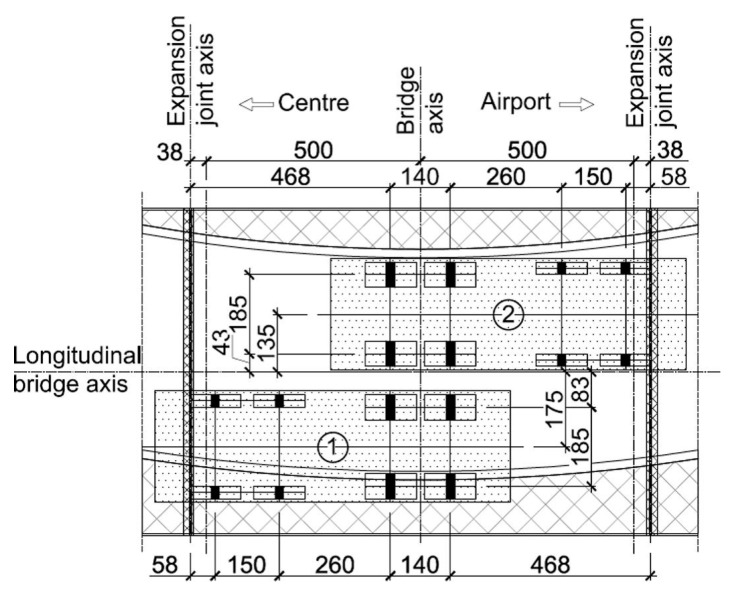
Scheme of the static load test with two trucks.

Each of the three tests included both static and dynamic load scenarios. Identical load schemes were employed in each test to facilitate a comparison of the behaviour of the FRP structure over time. For the static tests, two four-axle trucks, each with a nominal weight of 300 kN, were used, resulting in a total weight of 642.2 kN. Two loading configurations were implemented: the asymmetric scheme, which involved only one truck, and the symmetric scheme, where both trucks were positioned on the bridge (Figure 7). Dynamic tests were conducted with one truck passing over the bridge at three different velocities: 10 km/h, 20 km/h, and 30 km/h. Local road restrictions prevented the use of higher velocities during the dynamic tests. To enhance the dynamic effect, a 5 cm deep transverse threshold was incorporated, and two additional scenarios included sudden braking on the pavement, with truck velocities of 10 km/h and 15 km/h, respectively.

### 4.2. Instrumentation and Measurement Techniques

Strains, displacements, and dynamic parameters were recorded during each test using various instrumentation and measurement techniques. The most extensive tests, in terms of the number of measurements, were conducted in 2016 during the proof load (acceptance) tests. Vertical displacements were measured using LVDT sensors (DwyerOmega, Michigan City, IN, USA) at 12 discrete points on each girder’s bottom flange, specifically at three cross-sections: ¼, ½, and ¾ of the span length (L). Foil strain gauges were also positioned at these discrete points on the girder’s bottom and upper flanges, as well as in the deck slab between girders, across all three span sections. In total, 56 strain gauges were utilized in the static tests. The results of the conventional strain and displacement measurements from the initial proof load tests, along with the conclusions derived from them, were published in a separate paper [25].

We opted for distributed fibre optic sensing (DFOS) technology as the main structural health monitoring (SHM) system for Poland’s first all-composite bridge because of its internationally recognized benefits of fibre optic, along with its ability to measure strain across the entire bridge superstructure [31]. To measure a strain, we utilized the Luna OBR 4600 (Luna Innovations Inc., Roanoke, VA, USA) optical reflectometer that operates on linear Rayleigh scattering. We implemented single-mode telecom-grade optical fibres as sensors, setting virtual measurement sections at intervals of 10 mm to enable precise strain measurements every 10 mm along the optical fibres. The measured strain distributions can also be used to evaluate displacements, which are defined as changes in the shape of the laminates relative to their original form before loading. It is possible to determine strain profiles along the entire measuring length, identify the position of optical fibres concerning the neutral axis of the laminate (specifically, the bottom and top layers), assess the spatial resolution of the distributed fibre optic sensing system (which is 10 mm), and establish the boundary conditions. The algorithm for calculating displacements has been detailed in previous works by the authors [32,33]. Importantly, this algorithm does not require knowledge of material properties such as the modulus of elasticity, unlike the deflection equation. It can also be applied in scenarios involving large displacements. The algorithm is based solely on the geometrical analysis of a series of virtual gauges, where the base corresponds to the defined spatial resolution and the height represents the spacing between the optical fibres (lower and upper). Displacements are calculated for each gauge and subsequently summed to produce a comprehensive displacement profile along the entire measuring length.

**Figure 8:**
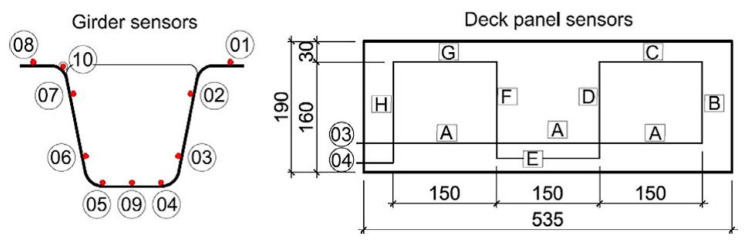
DFOS sensor’s layout for the girder and deck panel strains measurement (sensor no. 10 for thermal compensation only).

The fibre optic sensors were installed in the workshop after the FRP girders and deck panels were manufactured. A total of ten sensors, each measuring approximately 9.60 ± 0.10 m in length, were bonded with two-component epoxy glue on inside surfaces of two bridge girders (Figure 8). This configuration allows strain measurement at around 10,000 virtual discrete points along the girders. Sensor number 10 was separated in a polypropylene tube to monitor ambient temperature, which assists in adjusting for its impact on strain measurements. Fibre optic sensors were also installed on one of the bridge panels. Two sensors were positioned on the top surface of the panel (sensors 01 and 02), and an additional two were installed on the bottom surface (sensors 03 and 04). This setup allows for the measurement of strains in both the transverse and longitudinal directions of the panel.

The initial dynamic characteristics of the bridge were established in 2016 based on the measurement of girder and deck displacements by LVDT sensors (HBM, Inc., Marlborough, MA, USA) [25]. For each dynamic load scheme and at every LVDT measurement point, “time–displacement” plots were created. The dynamic coefficients for each scenario were calculated as the ratio of the maximum dynamic displacement to the maximum static displacement, which was determined by taking the average of the minimum and maximum displacements observed on the plot. The first natural frequency of the all-FRP composite superstructure was identified using the “time–displacement” plot from the dynamic load scheme with the transverse threshold. This was calculated as the ratio of the number of displacement peaks observed within a specific time, t_s_ (in seconds). In a recent test conducted in 2024, only the dynamic strain distributions, determined by DFOS system Luna ODiSI (Luna Innovations Inc., Roanoke, VA, USA) at 250 Hz frequency, were utilized to assess changes in the previously mentioned dynamic parameters: dynamic coefficients and the first natural frequency. The same dynamic load schemes used in the initial tests from 2016 were applied in this evaluation.

## 5. The Results of Subsequent Tests

### 5.1. Strains

**Figure 9:**
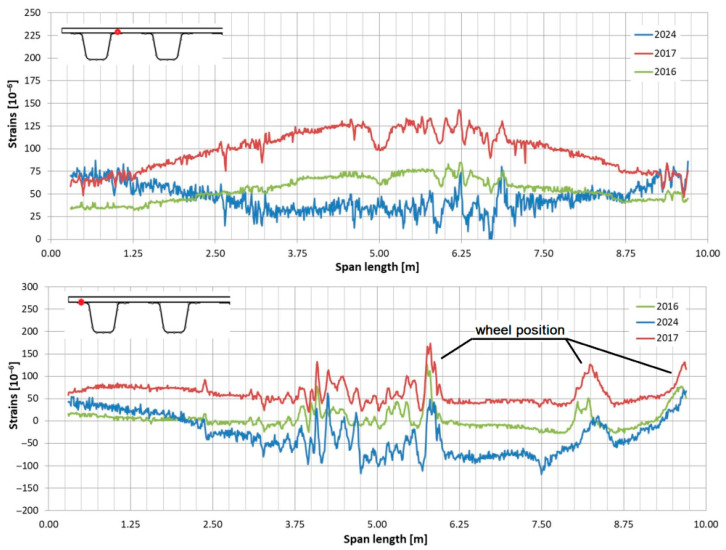
Strain distribution in the upper flange of the external girder measured by T01 (**top**) and T08 (**bottom**) sensors.

**Figure 10:**
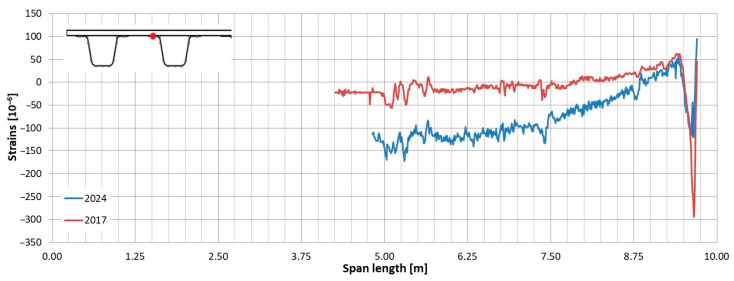
Strain distribution in the upper flange of the internal girder measured by T08 sensor (sensor T01 was damaged).

**Figure 11:**
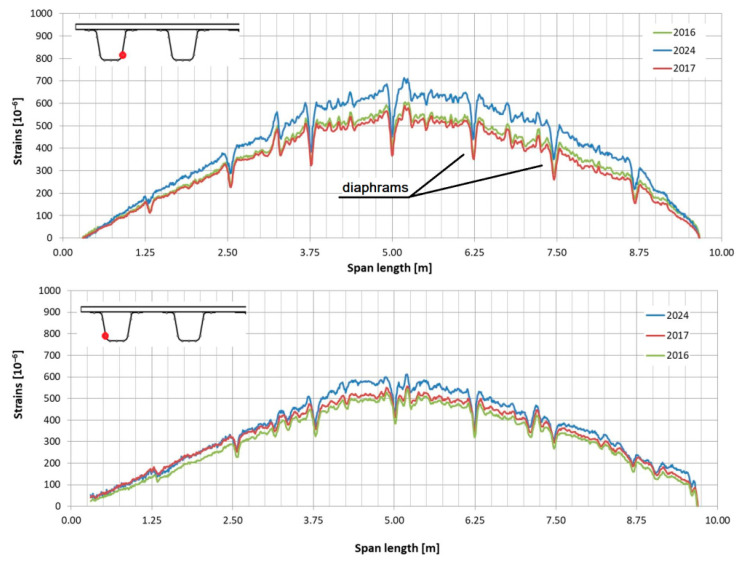
Strain distribution in the webs of the external girder measured by T03 (**top**) and T06 (**bottom**) sensors.

**Figure 12:**
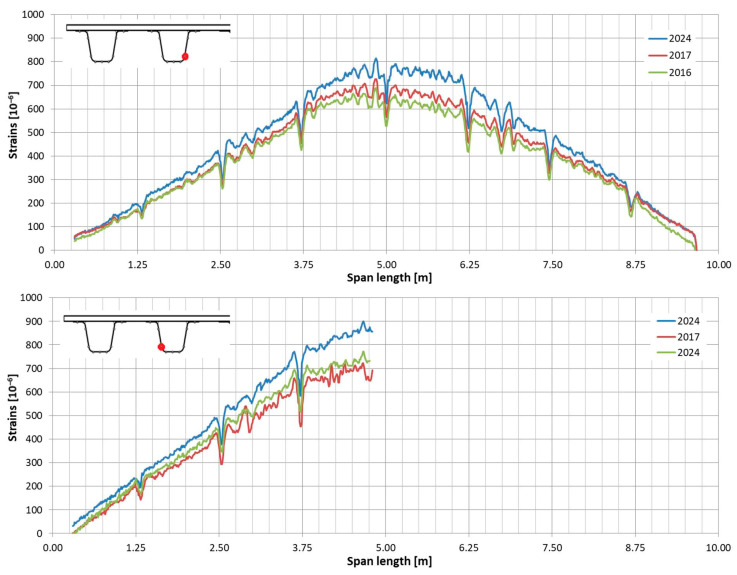
Strain distribution in the webs of the internal girder measured by T03 (**top**) and T06 (**bottom**) sensors (sensor T06 recorded strains up to half of the girder; beyond this point, it was damaged).

**Figure 13:**
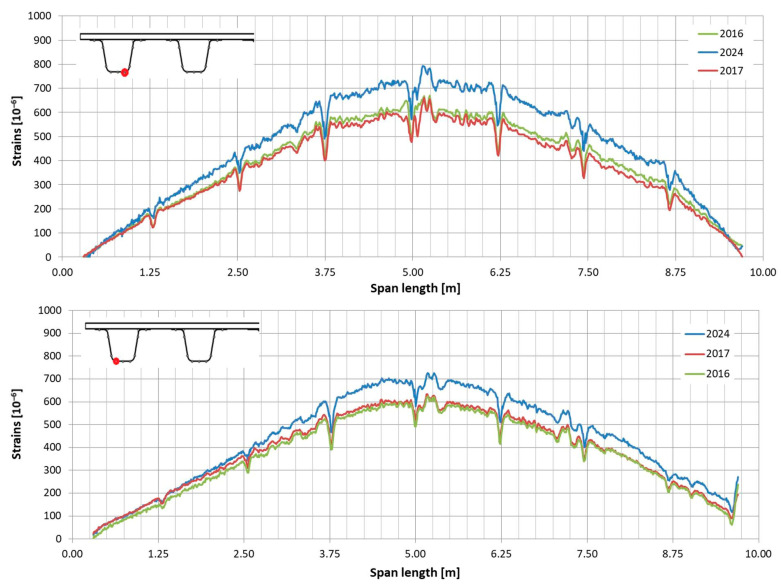
Strain distribution in the lower flange of the external girder measured by T04 (**top**) and T05 (**bottom**) sensors.

**Figure 14:**
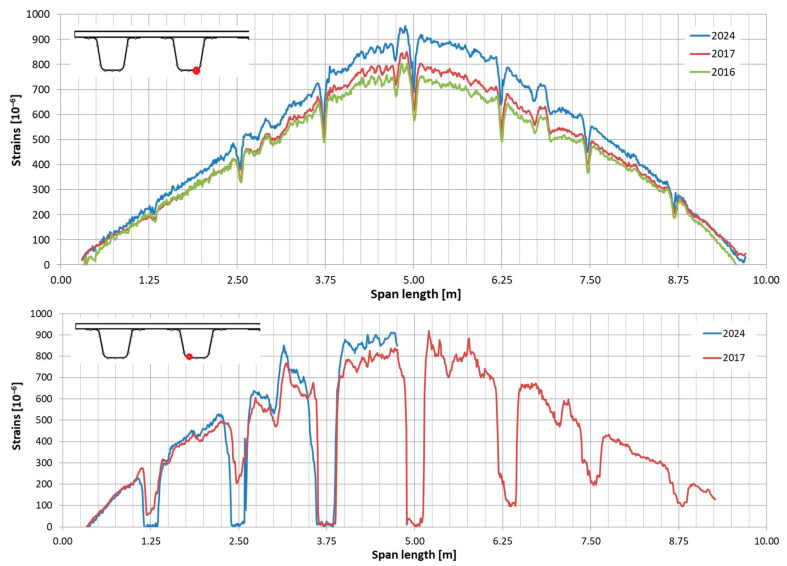
Strain distribution in the lower flange of the internal girder measured by T04 (**top**) and T05 (**bottom**) sensors (there was no record from T05 in 2016).

In the following plots (Figure 9, Figure 10, Figure 11, Figure 12, Figure 13 and Figure 14), we present the strain measurements obtained from selected fibre optic sensors located in two main girders: the external and internal girders. Positive values indicate tensile strains, while negative values represent compressive strains recorded at specific locations along the fibres. The plots display strains measured during all three tests to allow for better comparison. This comparison facilitates the evaluation of the performance of the FRP superstructure over the first eight years of service, taking into account the effects of traffic and environmental conditions on its structural behaviour. The qualitative comparison in the plots illustrates the differences in strain distribution during long-term service, which helps assess the current state of repair of the all-FRP composite bridge. In addition to the strain distributions displayed in the plots, Table 1 presents the maximum longitudinal stresses observed in the individual components of the girder (including the web and both flanges). This information allows for a quantitative comparison of the changes in strength utilization of each composite over an 8-year service period.

In comparing the strain distribution of FRP girders from three subsequent load tests, it can be concluded that there is a consistent, uniform increase in all strain values observed during the subsequent measurements. The plots also illustrate sudden changes in strain distribution, which are caused by local wheel pressures from the loading vehicles. This phenomenon is particularly noticeable in the upper flanges (Figure 9 and Figure 10). Additionally, another plot reveals abrupt strain changes in the girders’ sections, often occurring where geometric disturbances are present, such as diaphragms (Figure 11, Figure 12 and Figure 13). The presence of diaphragms is most evident at the T05 sensor, which was installed in the internal girder (Figure 14) to replace an original sensor damaged during the girder’s production. This sensor was positioned after the diaphragms were mounted, so it had to navigate around physical obstacles. Consequently, the location of the diaphragms is clearly visible on the plot.

In the lower sections of both girders, larger strains measured in 2024 are evident. This increase in strains indicates a change in the structure’s response to the same static load. The quantitative increase in the maximum recorded strain values in the bottom part of the girders is detailed in Table 2. Analysing the results in Table 2, the differences between 2016 and 2017 are minimal. The average strain difference in the tension section of the girder was 0.3% for the external girder and 1.6% for the internal girder. This suggests that the behaviour of the superstructure did not significantly change during the first year of service. In contrast, a comparison between the strains recorded in 2016 and those in 2024 reveals a notable increase. The average strain in the tension part of the external girder rose by 15.4%, while the internal girder experienced an increase of 17.7%. This indicates that after eight years of operation, there has been a significant change in the structure’s response to service loads. One possible reason for this change may be the damages observed in the all-FRP composite superstructure and the subsequent repairs made (see Section 3).

To evaluate the global behaviour of the all-FRP composite superstructure over time, we can analyse the transverse load distribution among the individual girders. Table 3 presents the ratio of strains between the external and internal girders over the subsequent years. This comparison shows that the difference in strain distributions between the two girders remained consistent over time. The average maximum strain values in the tension section of the internal girder compared to the corresponding strains of the external girder were 25.3%, 28.5%, and 26.6%, respectively, in 2016, 2017, and 2024. These changes are relatively insignificant, suggesting that despite the earlier mentioned damage and its subsequent repair, the overall behaviour of the superstructure has remained stable.

**Figure 15:**
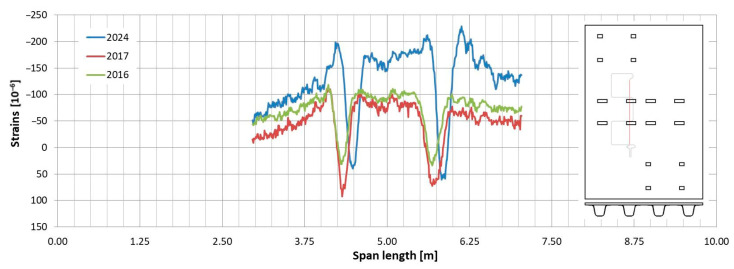
Strain distribution on the bottom facesheet surface of the deck panel measured in sensor “A”.

**Figure 16:**
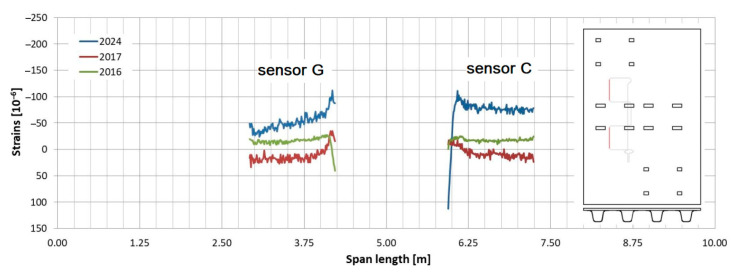
Strain distribution on the bottom facesheet surface of the deck panel measured in sensors “C” and “G”.

**Figure 17:**
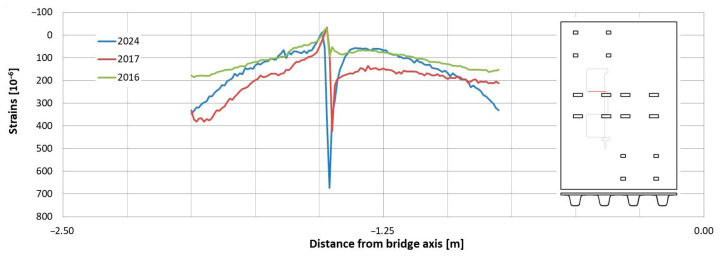
Strain distribution on the bottom facesheet surface of the deck panel measured in sensor “D”.

**Figure 18:**
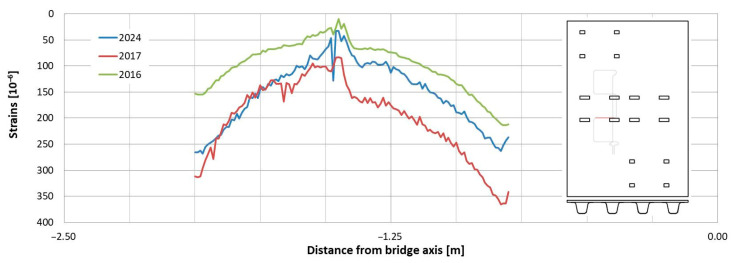
Strain distribution on the bottom face sheet surface of the deck panel measured in sensor “F”.

Observations regarding strain changes over time in the FRP composite deck panel reveal similar trends to those seen in girders. The longitudinal strains measured in 2024 are greater than those recorded in previous years (Figure 15 and Figure 16). Initially, during the bridge’s operation, the longitudinal strains remained near zero (as indicated by sensors “C” and “G” in Figure 16) or showed slight compression (as noted by sensor “A” in Figure 15). However, in the most recent measurements, a significant influence of compressive strains is evident in the deck panel, which functions as part of the composite girder. It indicates a shift in the position of the neutral axis of the composite girder over the evaluated service life of the bridge. In contrast to the longitudinal strains, the transverse strains on the bottom face sheet of the panel have remained relatively stable throughout the bridge’s service (Figure 17 and Figure 18). The variations in strain distribution observed in both plots, approximately 1.45 m from the bridge’s axis, are indicative of the edge of the panel support resting on the upper flanges of the girder. These variations result from the direct pressure exerted by these two elements, and the measuring sensor was positioned in the glued connection between them.

In addition to the strain distributions illustrated in the plots, Table 4 presents the extreme values of the longitudinal and transverse stresses in the bottom face sheet of the deck panel. This information allows for a quantitative comparison of how the strength utilization of the face sheet composite has changed over eight years of service.

### 5.2. Displacements

**Figure 19:**
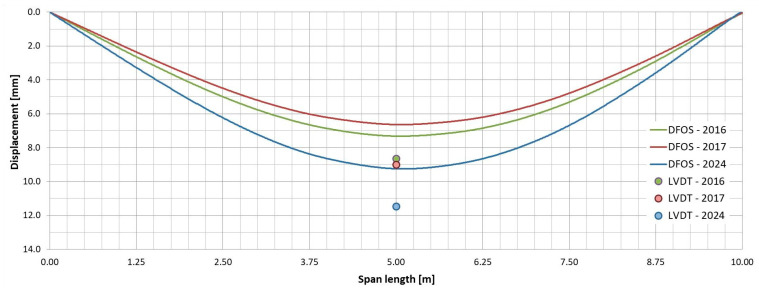
Displacement of external girder based on DFOS sensors (06–07 pair) and LVDT.

**Figure 20:**
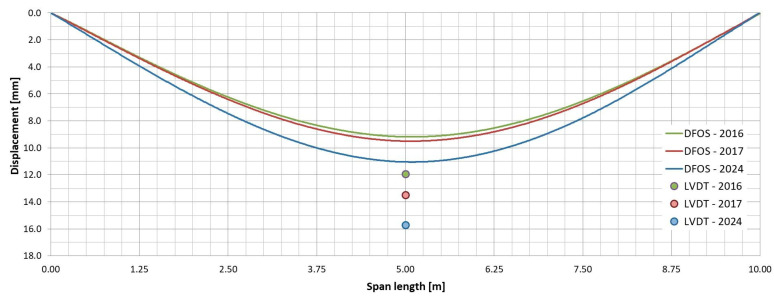
Displacement of internal girder based on DFOS sensors (03–04 pair) and LVDT.

As mentioned in Section 4.2, the displacements of the girders under the test load were measured using two methods: direct and indirect. The direct method employs LVDT sensors, a classic technique for measuring vertical displacements. In contrast, the indirect method relies on strain distributions measured by pairs of DFOS sensors, along with a calculation algorithm. Table 5 presents the deflections at the mid-span section as measured or determined by both methods. Additionally, Figure 19 and Figure 20 provide graphical representations of the displacements for selected pairs of DFOS.

The plots demonstrate that satisfactory accuracy in the deflection measurements obtained using DFOS system was achieved only for the external girder, with an accuracy of approximately ±15% (as referenced in [32]). In contrast, measurements taken from the internal girder exhibited a significant discrepancy, with deflections differing by approximately 23–30% from those measured with LVDT. These differences in measurements may arise from several factors. The indirect method, which is simpler to implement, relies on computational procedures that inevitably involve certain simplifying assumptions. Furthermore, strain distributions can be significantly affected by local factors, such as direct wheel pressure or the local bending of laminates. Another key consideration is that this method does not account for deflections caused by shear deformations in the bending girder. Shear deformation is critical when analysing FRP girders, particularly in scenarios where shear forces are substantial or where the girder’s geometry results in high shear stresses [34]. FRP materials typically exhibit lower shear moduli compared to their tensile moduli, leading to a larger share of the total deformation being attributed to shear, especially in cases of non-uniform bending. This is particularly relevant for all-FRP U-shaped girders with open cross-sections, which lack composite action.

### 5.3. Dynamic Parameters

**Figure 21:**
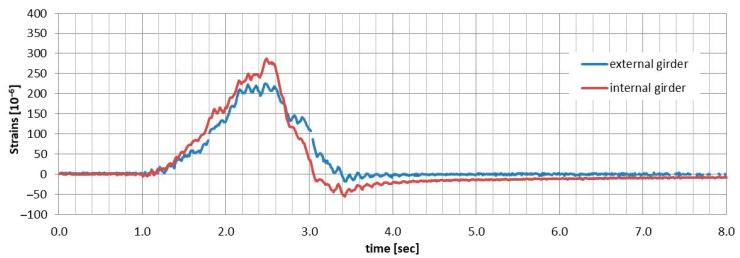
Dynamic strains in the bottom flanges of both girders during the passage of the truck at 20 km/h.

**Figure 22:**
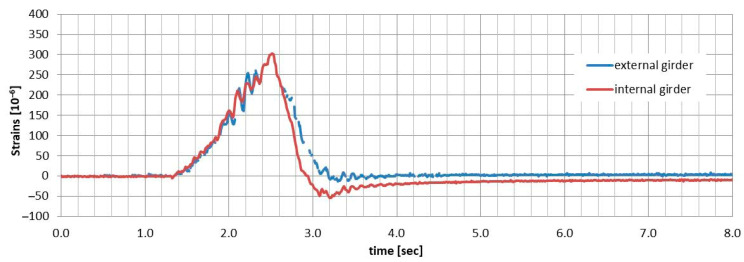
Dynamic strains in the bottom flanges of both girders during the passage of the truck at 30 km/h.

As previously mentioned, only the dynamic strain distributions obtained through DFOS system were used to evaluate the changes in two dynamic parameters: the dynamic coefficients and the first natural frequency. This assessment was part of a broader analysis of the bridge’s dynamic performance over time. To achieve this, the dynamic strain distribution over time during the passage of a loading truck was measured using selected optical fibres. Figure 21 and Figure 22 illustrate examples of “time–strain” plots for the mid-span of both external and internal girders, based on two selected load schemes (velocities) recorded with different optical fibres. The dynamic strain distributions obtained in this manner formed the foundation for creating the natural frequency spectrum of the superstructure, which was then used to determine the two dynamic parameters.

## 6. Long-Term Structural Performance of the All-FRP Composite Bridge

### 6.1. Strength and Load Carrying Capacity

The change in composite strains over time, measured using DFOS sensors, allows for a direct assessment of stress variations and the level of strength utilization in individual laminates of the composite superstructure. The stresses were calculated by multiplying the recorded strains by the modulus of elasticity, which for the applied laminates is 20.5 GPa [25]. The estimated stresses under the test load are summarized in Table 6. It is important to note that these values do not include the self-weight of the superstructure and equipment elements. Conversely, the ratio of the calculated stresses to the design strength of the composite indicates the extent to which the laminates’ strength is utilized under the test load. This ratio is commonly referred to as the failure index. The resulting failure index values for the individual elements are also presented in Table 6, using the design strength of the composites as determined in [25].

According to the compiled data, the maximum utilization of the laminates’ strength under the test load reaches up to 10% for girders and 8% for deck panels. The 10% value for girders remained unchanged throughout the bridge’s operation, while the 8% for deck panels increased fourfold compared to the initial proof load test. This change can be attributed to damage to the girders, which resulted in a temporary loss of composite action, and subsequent repairs. However, the test results confirmed that the repairs effectively restored the superstructure’s initial load-carrying capacity. Research has shown that the long-term behaviour of the first Polish all-FRP composite bridge is very similar to that of other structures of this type worldwide. The maximum recorded strains in subsequent tests were slightly higher than those observed during the initial proof load tests; however, they were still significantly lower than the approximate failure strains of the FRP composites used. This indicates a considerably greater load-carrying capacity, which results in a very high safety factor regarding the strength of the FRP composite materials.

### 6.2. Stiffness

**Figure 23:**
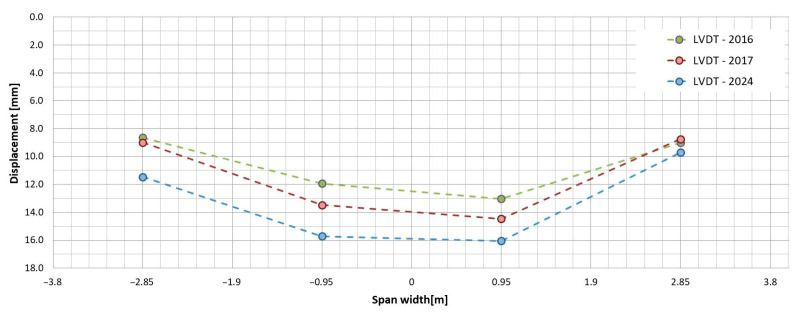
Transverse distribution of the mid-span girders’ deflections over time.

The change in stiffness of the all-FRP composite superstructure over time was evaluated based on mid-span girder deflections measured by an LVDT. The measured values are presented in Table 7, and the transverse distribution is illustrated in Figure 23. According to these measurements under the test load, the span deflection increased over time: after the first year of operation, it rose by an average of 6%, and after the subsequent 7 years, it increased by an additional 10%. This results in a total increase of 16% since the bridge was put into service. The initial increase is attributed to the structure’s adaptation to operating conditions, which is typical for all-FRP composite superstructures [14,18,20]. The second increase is due to damage sustained by the deck during operation and the loss of composite action between the deck panels and the girders. Consequently, the overall stiffness of the superstructure decreased, leading to increased deflections, particularly on the left side where the damage first occurred. If the repair of the damage is successful, further deflection should not occur. However, this will be assessed in the next phase of bridge monitoring scheduled for 2030. Until then, the bridge’s span structure, especially its repaired elements, will undergo visual inspections.

Additionally, it is important to note that the stiffness of all-FRP composite bridges can be influenced by temperature changes. However, the overall difference in maximum temperature response was less significant than the impact of a single truck load on the bridge [25]. Even though there has been a loss of stiffness after 8 years of operation, the deflection of the span under service loads—comparable to the test load—remains significantly lower than the limit set during the bridge design. During the tests conducted in 2024, the maximum deflection of the girder was recorded at 16.05 mm, which is only 54% of the permissible deflection limit of L/300, equating to 30 mm [25].

### 6.3. Dynamic Performance

Due to space limitations in this paper, the evaluation of the time-dependent dynamic performance of the all-FRP composite bridge has been restricted to two parameters: the first natural frequency and the dynamic coefficients for the applied load schemes. These parameters were determined based on the dynamic strain distribution, as illustrated in Figure 21 and Figure 22. Mean values for both parameters were calculated using measurements from two optical fibres located in the bottom flange of each girder (sensors 04 and 05, as shown in Figure 8). The parameters obtained from DFOS measurements were then compared to the initial values recorded in 2016, which were based on displacement measurements taken with LVDT sensors.

Regarding the first natural frequency, the current value of 9.1 Hz is approximately 10% lower than the initial value of 10.1 Hz, which was determined in 2016. This decrease is comparable to the experimentally determined reduction in superstructure stiffness of 16% (Table 7) and is attributed to damage and subsequent repairs made to the bridge. Despite this reduction, the recently determined first natural frequency of the all-FRP composite span remains significantly higher (and thus better) than the recommended minimum value of 3 Hz according to Polish bridge requirements. Additionally, the slight reduction in the bridge’s stiffness has led to a minor increase in the dynamic coefficient, which averages +3.6% compared to the initial value from 2016 (Table 8). However, this current value of 1.13 is still considerably lower than those determined for other bridges of this type [14,19,20]. It is also important to note that the dynamic coefficient continues to show no dependence on truck velocity; it remains essentially constant. This stability may be attributed to the bridge’s location, where it was not possible to achieve higher speeds during the dynamic load test. Therefore, this conclusion may not be applicable to all FRP composite bridges.

The remaining dynamic parameters, such as the logarithmic decrement and damping ratio, along with their changes over time (which are not reported here), further confirmed that the all-FRP composite bridge was performing well in service and showed no structural problems. Since the dynamic characteristics of lightweight all-FRP composite bridges are crucial for their operation, monitoring these characteristics is the most effective and cost-efficient approach. Therefore, the dynamic tests conducted on the all-FRP composite bridge in Rzeszów and their results will be discussed in a separate paper that will be published elsewhere.

### 6.4. Effectiveness of Repair

**Figure 24:**
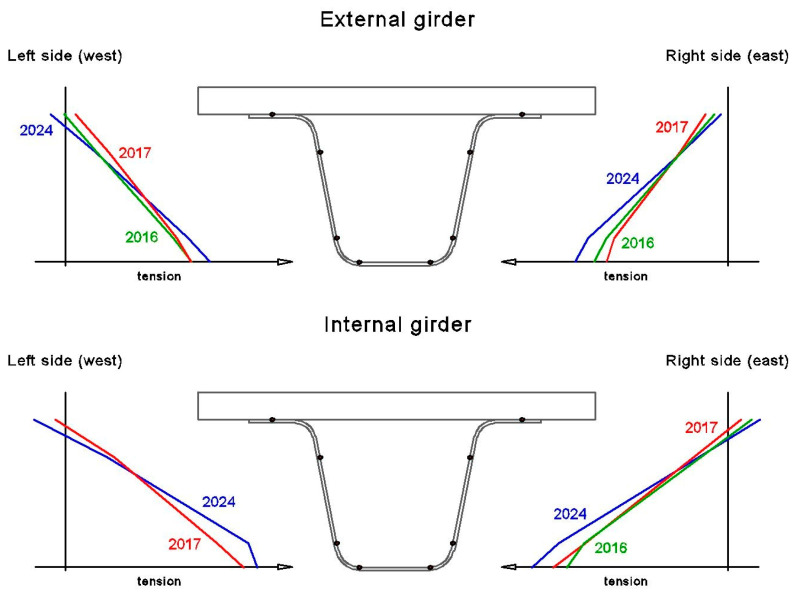
Longitudinal strain through the depth of mid-span girders’ cross-sections.

During the operation of the bridge, damage was observed that led to the temporary disintegration of the girder cross-section. This issue arose from a lack of connection between the girder body and the deck panels. The momentary absence of composite action likely reduced the load-carrying capacity of the superstructure. To address this, a detailed analysis was conducted to assess changes in the position of the neutral axis of the girders over time. The position of the neutral axis was determined experimentally, and the strain values obtained at the mid-span cross-section of both girders are presented in Table 1 and illustrated graphically in Figure 24.

The position of the neutral axis of both girders changed over time (Figure 24). The highest position of the neutral axis was recorded in 2017, with an average change of approximately 50 mm due to damage to the deck and a partial loss of the composite action between the girders. However, this change in the neutral axis position was not significant given that the girder depth is 840 mm, meaning it had minimal impact on the load-carrying capacity of the girders. Following the repair of the deck and the restoration of the composite action, the neutral axis dropped again in 2024, returning to its initial position from 2016. This outcome confirms the effectiveness of the superstructure repair.

## 7. Conclusions and Future Perspectives

The article discusses the technical condition and long-term performance of the first Polish all-FRP composite bridge after eight years of operation. A visual inspection was conducted to assess the bridge’s condition. Additionally, three identical tests were performed under static and dynamic loads in 2016, 2017, and 2024. These tests measured changes in strains, displacements, and dynamic parameters of the all-FRP composite superstructure throughout its operation. The observed changes in these key values and parameters facilitated both a quantitative and qualitative evaluation of the bridge’s long-term performance, highlighting alterations in strength, load-carrying capacity, stiffness, and dynamic behaviour. The effectiveness of the bridge’s repairs was also assessed.

Based on the analysis of the all-composite bridge’s structural behaviour and monitoring data, the following conclusions can be drawn:

- The first all-FRP composite bridge in Poland has shown satisfactory structural integrity and durability over an operational period of eight years. The monitoring data indicates that there have been minimal changes in strength, stiffness, and dynamic performance over the long term. Periodical field load testing has confirmed that this all-FRP composite bridge has maintained reliable performance during the years it was monitored, and it can be considered a dependable structure.
- The quality of the adhesive joints between the girders and the deck panels was inadequate, which led to a slight decrease in the bridge’s performance. Local damage to these connections caused a 16% reduction in the span’s stiffness and a deterioration in its dynamic characteristics, including a 10% reduction in the first natural frequency and a 3.6% increase in the dynamic coefficient. However, these negative changes did not compromise the bridge’s safety or its functionality according to the design requirements. An effective repair was completed, restoring the bridge to its full operational efficiency.
- The presented strain measurement system (DFOS) has demonstrated itself as an efficient and cost-effective approach for monitoring FRP bridges. It allows for the assessment of changes in the bridge’s load-carrying capacity, stiffness, and dynamic characteristics while it is in service.

Future research should emphasize the further long-term observation of the bridge’s performance under service loads. To better analyse the bridge’s behaviour, the dynamic characteristics of the superstructure will be utilized more extensively. Monitoring these characteristics is not only cost-effective but also provides more informative and reliable data compared to simple strain and displacement measurements. Additionally, further development of the distributed fibre optic sensing system is anticipated through ongoing research to enhance the accuracy of dynamic measurements and apply them for evaluating dynamic performance. Lastly, in the subsequent measurement phases, the effects of temperature and creep on the behaviour of the all-FRP composite structure will be assessed.

## Figures and Tables

**Table 1:** Maximum longitudinal strains in the girders’ composites (mid-span section).

Girder/Component		Strains [με]											
		2016				2017				2024			
External	Upper flange	85	128 *	N/A *	N/A	143	174 *	N/A	N/A	80	70 *	N/A	N/A
	Web	S/F	606	325 *	543	340 *	589	368 *	558	300 *	712	393 *	614
	Bottom flange	670	626	721	N/A	661	633	712	N/A	793	726	808	N/A
Internal	Upper flange	S/F	S/F	N/A	N/A	−62	−50 **	N/A	N/A	−179	−151 **	N/A	N/A
	Web	S/F	689	S/F	772 **	S/F	727	334 *	724 **	S/F	815	326 *	899 **
	Bottom flange	804	S/F	698	N/A	850	940	784	N/A	953	911 **	839	N/A

N/A—Not available (no sensor at this location or sensor damaged at production stage). S/F—sensor failure (one-time problem with sensor readings). * Measured at a cross-section 70 cm away from the mid-span due to the direct load of the truck’s wheel. ** The sensor only covered half of the girder.

**Table 2:** The changes in maximum strains over 8 years for individual elements of the girders.

Sensor	Strain Changes *							
	External Girder				Internal Girder			
	2017 vs. 2016		2024 vs. 2016		2017 vs. 2016		2024 vs. 2016	
	Absolute	Relative	Absolute	Relative	Absolute	Relative	Absolute	Relative
	[με]	[%]	[με]	[%]	[με]	[%]	[με]	[%]
Bottom flange								
T5	+7	+1.1	+100	+15.9	-	-	-	-
T9	−9	−1.3	+84	+12.1	-	-	-	-
T4	−10	−1.4	+123	+18.3	+46	+5.7%	+149	+18.5
Web								
T6	+15	+2.8	+71	+13.1	−48	−6.2%	+127	+16.4
T3	−17	−2.9	+107	+17.6	+37	+5.4%	+125	+18.2
Average of all above sensors		−0.3		+15.4		+1.6%		+17.7

* A positive value means higher values of tensile strains.

**Table 3:** The differences in maximum strains in internal and external girders under the subsequent loads.

Sensor	Maximum Strain Differences: Internal vs. External Girder (Bottom Flange) *					
	2016		2017		2024	
	Absolute	Relative	Absolute	Relative	Absolute	Relative
	[με]	[%]	[με]	[%]	[με]	[%]
Bottom flange						
T5	-	-	203	32.1	185	25.4
T4	134	20.0	189	28.6	160	20.2
Web						
T6	229	42.2	166	29.7	285	46.4
T3	83	13.7	138	23.4	103	14.5
Average of all above sensors		25.3		28.5		26.6

* A positive value means higher values of tensile strains.

**Table 4:** Maximum/minimum longitudinal and transverse strains in the deck panel’s face sheet.

Panel Direction	Sensor	Strains [με]		
		2016	2017	2024
Longitudinal	A	34/−118	93/−110	61/−229
	C	−1/−25	25/−18	112/−111
	E	59/−129	94/−127	191/−270
	G	41/−28	34/−35	−24/−112
Transverse	B	108/45	207/102	135/84
	D	185/−28	423/−34	674/−13
	F	214/10	365/83	268/33
	H	67/1	168/50	119/17

**Table 5:** The mid-span deflections based on different measurement methods.

Girder	Pairs of Sensors	Mid-Span Deflection [mm]		
		2016	2017	2024
		DFOS (indirect, calculated)		
External	2–3	-	5.82	9.66
	6–7	7.32	6.64	9.25
	1–4	7.42	6.16	9.36
	5–8	7.99	7.27	10.27
	3–4	6.18	5.86	7.97
	5–6	9.15	8.02	11.97
	1–2	-	6.98	9.50
	7–8	8.67	7.97	11.16
	Average of all above sensors	7.79	6.84	9.89
		LVDT (direct, measured)		
		8.64	9.00	11.46
		Ratio DFOS/LVDT [%]		
		90.1	76.0	86.3
Internal		DFOS (indirect, calculated)		
	1–4	-	10.43	10.91
	3–4	9.18	9.50	11.05
	Average of all above sensors	9.18	9.97	10.98
		LVDT (direct, measured)		
		11.94	13.47	15.72
		Ratio DFOS/LVDT [%]		
		77.0	74.0	70.0

**Table 6:** Maximum stresses and failure indices in the particular FRP superstructure elements.

Bridge Element	Component/Type		Design Strength [MPa]	Stress [MPa]			Failure Index		
				2016	2017	2024	2016	2017	2024
External girder	Solid	UF	184	1.4	2.2	1.7	1	1	1
	Sandwich	W		11.8	11.0	13.6	6	6	7
	Solid	BF		13.0	12.2	14.8	7	7	8
Internal girder	Solid	UF	184	-	1.2	3.2	-	1	2
	Sandwich	W		14.8	14.6	17.7	8	8	10
	Solid	BF		15.7	17.2	19.0	9	9	10
Deck panel	Solid	BS	184	4.4	8.7	13.8	2	5	8

**Table 7:** The mid-span girders’ deflections in the subsequent load tests.

Girder	2016	2017		2024	
	Deflection	Deflection	Increase to 2016	Deflection	Increase to 2016
	[mm]	[mm]	[%]	[mm]	[%]
D1	8.64	9.00	4	11.46	27
D2	11.94	13.47	13	15.72	17
D3	13.04	14.47	11	16.05	11
D4	9.02	8.76	−3	9.72	11
Average increase			6		16

**Table 8:** Initial and recent dynamic coefficients (or impact factors).

Truck Velocity [km/h]	2016	2024	
	Initial Dynamic Coefficients	Current Dynamic Coefficients	Increase
			2024 vs. 2016 [%]
10	1.08	1.12	+3.7
20	1.10	1.15	+4.5
30	1.10	1.13	+2.7
Average	1.09	1.13	+3.6

## Data Availability

The data presented in this study are available on request from the corresponding author.
